# 

**Published:** 1888-05

**Authors:** 


					﻿Vol. IX.	MAY, 1888.	No. 5?
THE
IndciimdcniPrarhüonfT
A. Iokthiy iOWM
DEVOTED TO
DENTAL AND ORAL SCIENCE.
W. C. BARRETT, M. D„ D. D. S.,
EDITOR.
The Hew York Dehtal Johrkal Association
PUBLISHERS,
No. 33 West 47th. Street, New York,
AND
No. SOS .Franklin St., Buffalo, N. Y.
$2.50 Per Annum in Advance, .... Single Copies, 25 Cents.
Entered at the Post-Office. Buffalo. N. Y.. as Second-Class matter.
				

